# Genetic diversity in natural populations of *Jacaranda decurrens* Cham. determined using RAPD and AFLP markers

**DOI:** 10.1590/S1415-47572010005000068

**Published:** 2010-09-01

**Authors:** Bianca W. Bertoni, Mariana P. de C. Telles, Milena G. Malosso, Simone C. Z. Torres, José O. Pereira, Mirian V. Lourenço, Suzelei de C. França, Ana M. S. Pereira

**Affiliations:** 1 Unidade de Biotecnologia Vegetal, Universidade de Ribeirão Preto, Ribeirão Preto, SPBrazil; 2 Laboratório de Genética e Biodiversidade, Instituto de Ciências Biológicas, Universidade Católica de Goiás, Goiânia, GOBrazil; 3 Programa de Pós-Graduação em Biotecnologia, Universidade de Ribeirão Preto, Ribeirão Preto, SPBrazil; 4 Centro de Apoio Multidisciplinar, Universidade Federal de Amazônia, Manaus, AMBrazil

**Keywords:** Bignoniaceae, carobinha, Cerrado, germplasm bank, preservation

## Abstract

*Jacaranda decurrens* (Bignoniaceae) is an endemic species of the Cerrado with validated antitumoral activity. The genetic diversity of six populations of *J. decurrens* located in the State of São Paulo was determined in this study by using molecular markers for randomly amplified polymorphic DNA (RAPD) and amplified fragment length polymorphism (AFLP). Following optimization of the amplification reaction, 10 selected primers generated 78 reproducible RAPD fragments that were mostly (69.2%) polymorphic. Two hundred and five reproducible AFLP fragments were generated by using four selected primer combinations; 46.3% of these fragments were polymorphic, indicating a considerable level of genetic diversity. Analysis of molecular variance (AMOVA) using these two groups of markers indicated that variability was strongly structured amongst populations. The unweighted pair group method with arithmatic mean (UPGMA) and Pearson's correlation coefficient (RAPD -0.16, p = 0.2082; AFLP 0.37, p = 0.1006) between genetic matrices and geographic distances suggested that the population structure followed an island model in which a single population of infinite size gave rise to the current populations of *J. decurrens*, independently of their spatial position. The results of this study indicate that RAPD and AFLP markers were similarly efficient in measuring the genetic variability amongst natural populations of *J. decurrens.* These data may be useful for developing strategies for the preservation of this medicinal species in the Cerrado.

## Introduction

The study of population and genetic diversity is a complex subject that involves the analysis of DNA sequences, gene adaptability, inter-individual variation and speciation, and an understanding of the interactions among organisms that compose communities. Furthering knowledge about genes, individuals, species and communities provides an ever greater understanding of biodiversity and, consequently, allows the development of the most adequate strategies for environmental preservation. Molecular markers provide an important tool for assessing the genetic variability and structure of natural populations, and for studying biodiversity in general (Frankham *et al.*, 2002). They also provide a basis for developing programs to protect flora threatened with extinction.

The random amplification of polymorphic DNA (RAPD) provides markers that can be used to identify and discriminate genotypes, in addition to providing a means for assessing phenotypic expression and phylogenetic relations in the germplasm under study (Ferreira and Grattapaglia, 1998). Similarly, amplified fragment length polymorphism (AFLP) allows the study of genetic polymorphisms in populations, with the advantage that multiple reaction loci can be detected with high reproducibility (Jain *et al.*,1994; Hill *et al.*, 1996). Polymorphisms detected by AFLP generally indicate Mendelian inheritance and may be used to study kinship and genetic variability within and between populations. AFLP is also a robust tool for DNA fingerprinting of genomes (Vos *et al.*, 1995).

*Jacaranda decurrens* is a medicinal plant endemic to the Cerrado. One of its components, ursolic acid, is considered to have antitumoral activity (Varanda *et al.*, 1992; Subbaramaiah *et al.*, 2000). Indiscriminate collection and frequent deforestation of the Cerrado have damaged the species diversity of this biome and accelerated the ever increasing risk of extinction. In this study, we used RAPD and AFLP markers to calculate the genetic distances among populations of *J. decurrens* in the Cerrado of the State of São Paulo, in southeastern Brazil.

## Material and Methods

**Figure 1:**
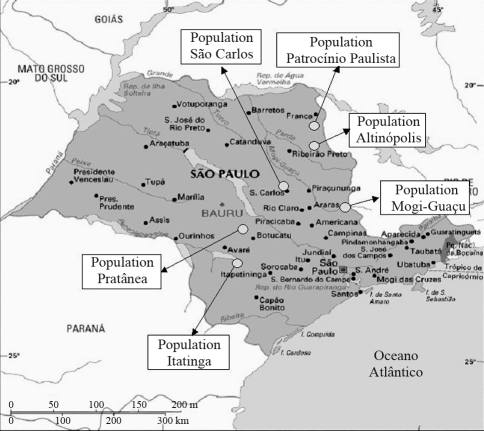
Geographic location of the *J. decurrens* populations studied in this work.

Ninety individuals of *J. decurrens* from six populations were used in this study. The collection sites were within the species distribution range in the State of São Paulo (SP) (Figure 1). The areas of collection at each site were randomly chosen to a precision of 10 m and their precise location was determined with a global positioning satellite (GPS) system. Distances among populations were determined by using the computer program GPS TRACKMAKER 11.7 (Ferreira, 2001) and are shown in Table 1. Exsiccates of all accesses were prepared and deposited in the Herbarium of Medicinal Plants at the University of Ribeirão Preto (HPM - UNAERP, Ribeirão Preto, SP, Brazil). Genomic DNA was isolated from young leaves using the protocol proposed by Doyle and Doyle (1987).

###  Acquisition of molecular data

Samples of *J. decurrens* DNA were initially evaluated with 110 primers for RAPD (Operon Life Technology and Biosynthesis Incorporated), 30 of which were selected for further investigation. The reactions were optimized by the amplification protocol of Ferreira and Grattapaglia (1998), and the amplification products (bands) were separated by electrophoresis in 1.5% (w/v) agarose gels that were subsequently stained with ethidium bromide. DNA markers (100 bp) were included in each electrophoretic run. All of the reagents were from Amersham Biosciensces. The gels were photographed under ultraviolet light (Image Master VDS, equipment, Pharmacia Biotech) and only reproducible bands were used in subsequently analyses. Weak bands for amplification fragments were excluded. Control samples containing all of the reaction products except DNA were used to confirm the absence of self-amplification and contaminants.

Of 30 RAPD primers selected, 10 showed a high degree of polymorphism and better band intensity (Table 2). After optimizing the parameters of the chain polymerase reaction (PCR), the following protocol was used for RAPD of *J. decurrens* (final reaction volume = 30 μL): 3.0 μL of Tp 10X buffer, 3.0 μL of 2.5 mM dNTPs, 2.4 μL of 25.0 mM MgCl_2_, 5.0 μL of primer (10 ng/μL), 0.3 μL of *Taq* DNA polymerase (5 U/μL) and 4.0 μL of sample DNA (5 ng/μL). The amplifications were done in a thermocycler using the following conditions: 2 cycles at 94 °C for 2 min, 1 cycle at 37 °C for 1 min, 1 cycle at 72 °C for 2 min, and 33 cycles at 94 °C for 10 s, 40 °C for 20 s (annealing temperature) and 72 °C for 2 min.

AFLP was done using a protocol adapted from Vos *et al.* (1995). Genomic DNA (200 ng) was digested with the restriction enzymes *Eco*RI and *Mse*I at 37 °C for 3 h in a model PTC-100 thermocycler (MJ Research Inc). The resulting fragments were ligated to specific adapting sequences complementary to the extremities cleaved off by the two enzymes in a reaction done at 23 °C for 3 h.

The ligated fragments (50 μL) were pre-amplified with primers containing a selective base, *Eco*-A/*MseI*-A and the PCR products of this pre-amplification were used as a template for the selective amplification in which primers with three selective nucleotides were added to the 3' extremity of the primers. Four combinations that were helpful in detecting polymorphisms were used, namely, *Eco*-ATC/*MseI*-ATC, *Eco*-ATT/*MseI*-ATG, *Eco*-ATG/*MseI*-AGT and *Eco*-AGT/*MseI*-ATT.

The components of the pre-amplification reaction were: 1 μL of *Eco*RI-primer with one selective base (25 ng/μL), 1 μL *MseI* + (primer with one selective base) (25 ng/μL), 0.8 μL of 2.5 mM dNTPs, 2 μL of 10 X buffer B (Promega), 1.2 μL 25 mM MgCl_2_, 0.6 μL *Taq* DNA polymerase (5 U/μL) (Promega) and 2 μL of the ligated DNA. The reaction conditions were as follows: 1) 94 °C for 2 min, 2) 94 °C for 1 min, 3) 56 °C for 1 min, 4) 72 °C for 1 min, and 5) 72 °C for 5 min; steps 2 to 4 were repeated 26 times. The amplification products were subsequently diluted four-fold and stored at -20 °C.

For selective amplification by AFLP-PCR the reaction mixture contained: 1 μL of *Eco*RI + (primer three selective base) (25 ng/μL), 1.2 μL *MseI* + (primer with three selective bases) (25 ng/μL), 0.4 μL of 2.5 mM dNTPs, 2 μL 10 X buffer B (Promega), 1.2 μL of 25 mM MgCl_2_, 0.2 μL of *Taq* DNA polymerase (5 U/μL) (Promega) and 1.5 μL of pre-amplified DNA. The reaction conditions were as follows: 1) 94 °C for 2 min, 2) 94 °C for 30 s, 3) 65 °C for 30 s, 4) 72 °C for 1 min, 5) 94 °C for 30 s, 6) 56 °C for 30 s, 7) 72 °C for 1 min and 8) 72 °C for 2 min; steps 2 to 4 were repeated 12 times and steps 5 to 7, 23 times. The amplification products were stored at -20 °C.

The AFLP products were separated by electrophoresis in denaturing 6% polyacrylamide gels in TBE 1X buffer for 2 h at 50 °C and 80 W constant voltage. The gels were stained with silver nitrate solution and developed in sodium carbonate, as described by Creste *et al.* (2001).

###  Statistical analyses

The binary data obtained from RAPD and AFLP were used to estimate allele frequencies based on a correction proposed by Lynch and Milligan (1994). A descriptive analysis of total variability was obtained by calculating the percentage of polymorphic loci and Nei's (1978) diversity index. AMOVA was used to separate the total genetic variance into its between- and within-population components; this allowed an assessment of variability, as proposed by Excoffier *et al.* (1992). The programs TFPGA (Miller 1997), AMOVA-PREP 1.01 (Miller 1998) and WINAMOVA 1.04 (Excoffier 1992) were used for these analyses. Nei's genetic distances (Nei, 1978) were used in a UPGMA grouping analysis to assess the genetic divergence among the populations studied.

## Results

**Figure 2:**
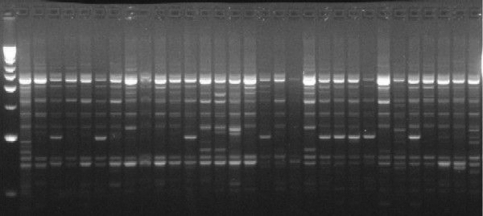
Profile of RAPD bands obtained with the primer CAG GCC CTT C for natural populations of *J. decurrens*. Lane 1 - molecular weight marker (100 bp ladder).

**Figure 3:**
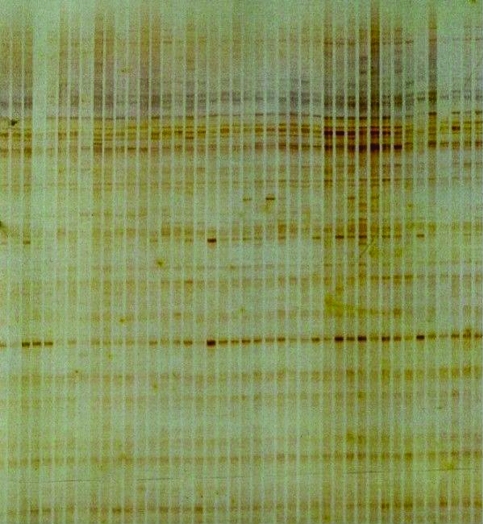
Profile of AFLP bands obtained with the primer combination E-ATG+M-AGT for natural populations of *J. decurrens*.

The ten primers chosen produced 78 bands in the six populations, with most (69.23%) of the bands being polymorphic. The minimum and maximum number of bands per primer was, respectively, 3 and 12 (Table 2). Figure 2 shows a typical example of the polymorphism detected for the primer Operon A-18. The four combinations of AFLP markers used yielded 205 bands, 46.34% of which were polymorphic. The minimum and maximum number of bands per primer was, respectively, 29 and 73 (Table 3). An example of the polymorphism detected with the E-ATG+M-AGT combination is shown in Figure 3.

###  Genetic variability within and between populations

The genetic characteristics of the six populations based on the allele frequencies (assuming Hardy-Weinberg equilibrium for the 205 loci studied) are shown in Table 4. The percentage of loci polymorphic for RAPD and AFLP among the 90 individuals examined was 69.23% and 46.34%, respectively. The population at Altinópolis had the highest percentage of polymorphic loci for the two markers (62.82% for RAPD and 33.17% for AFLP), followed by the populations at Itatinga (23.08% and 28.29%, respectively) and Pratânea (19.23% and 26.83%, respectively).

AMOVA based on the RAPD and AFLP markers indicated that 69.69% and 73.66% of the genetic variability was in the intra-population component. The PHI_ST_ value was 0.303 for RAPD, while for AFLP it was of 0.263 (p < 0.001); both markers indicated a highly significant structuring of the genetic variability in these populations (Table 5).

**Figure 4:**
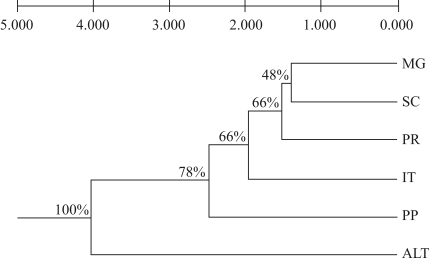
Pattern of genetic divergence among six populations of *J. decurrens* in the State of São Paulo based on the analysis of RAPD and AFLP markers. The relationships were assessed by the UPGMA method based on Nei's genetic distances (Nei,1978).

The UPGMA dendrogram based on Neis genetic distances indicated high variability among the populations, with that from Altinópolis showing the greatest divergence (0.0403) while significant similarity was observed among the populations from Mogi-Guaçú and São Carlos (0.0139) (Figure 4).

The spatial pattern determined by the Mantel test based on 5000 permutations was 0.37 (p = 0.1006) and -0.16 (p = 0.2082) for AFLP and RAPD, respectively. This finding indicated that there was no significant matricial correlation between geographic and genetic distances. These results clearly indicate that geographic distance alone does not explain the spatial pattern of genetic diversity among the populations. They also show that there is no spatial structuring of the genetic variability for AFLP and RAPD markers in the *J. decurrens* populations studied here. Both findings support a model of partially isolated islands that evolved independently in genetic space.

## Discussion

This is the first study to use RAPD and AFLP molecular markers to examine the genetic variability of natural populations of *J. decurrens* in the State of São Paulo. The high variability reported here indicates that these markers are useful for analyzing the genetic constitution of the six populations that were studied; this conclusion was supported by the number of polymorphic bands observed (Tables 2  and 3), by the allele frequencies (assuming Hardy-Weinberg equilibrium) (Table 4) and by AMOVA (Table 5).

Many studies with RAPD and AFLP markers have confirmed the efficacy of these techniques in assessing the variability and genetic structure of populations in a variety of plants, including cocoa (Russell *et al.*, 1993), *Azadirachta indica* (Singh *et al.*, 2002)*, Saccharum* spp. (Lima *et al.*, 2002), rice (Ge *et al.*, 1999), mahogany (Gillies *et al.*, 1999), pepper (Wadt and Kageyama, 2004), *Ilex paraguariensis* (Gauer and Cavalli-Molina, 2000)*, Hypericum perforatum* (Arnholdt-Schmitt, 2000), *Digitalis minor* (Sales *et al.*, 2001) and *Tabebuia impetiginosa* (Ciampi *et al.*, 2003), the last four species being medicinal plants.

Variation in the allele frequency between populations (F_ST_, proposed by Wright) reflects the probability that two random genes from two populations are identical by descent (Futuyma, 1992). Population subdivision may affect the allele frequencies and consequently the proportion of genetic variability between populations (Solferini and Selivon, 2001). Previous analyses of genetic variability between and within *J. decurrens* populations estimated the variation in PHI_ST_ for RAPD and AFLP markers to be 0.303 and 0.263, respectively, *i.e.*, greater than the upper limit of 0.25 proposed by Wright (Solferini and Selivon, 2001); these elevated values indicate a high degree of high population structuring.

The greatest genetic variability in the *Jacaranda* populations (Table 5) occurred within populations (69.69% for RAPD and 73.66% for AFLP). A similar degree of structures was reported for *Tabebuia impetiginosa*, another Bignoniaceae native to human-modified habitats; in this case, the genetic variability within populations was also greater than between them (~94% greater) (Ciampi *et al.*, 2003).

The dispersion of *J. decurrens* and *T. impetiginosa* seeds is completed by wind because the seeds are wispy and have membranous husks that favor their migration over large distances. According to Loveless and Hamrick (1984), seed dispersion by wind increases variation within populations. Conversely, depending on the wind velocity and the seed characteristics, migration over long distances can avoid population divergence. *J. decurrens* shows an ecological interaction with fire via a phenomenon known as pyrophytic adaptation in which fire, in addition to opening fruits that are close to the soil surface because of their weight, also eliminates the barrier created by leaves and small twigs of the plant itself; fire also reduces the interference of neighboring small plants on jaracanda seed dispersal (Coutinho 1977). In the absence of burnings, seeds are prevented from being taken over long distances by the wind; consequently, variability between populations increases and could explain the marked variation seen here between populations of *J. decurrens*.

There was no obvious relationship between the genetic and geographic distances among the populations studied here. This finding suggests that the structure of the *J. decurrens* populations in this study followed the island model proposed by Wright (Solé-Cava, 2001) in which the differentiation between populations does not depend on the distance that separates them. A single population of infinite size or other islands could have given rise to the present *J. decurrens* populations, without any particular relationship to their spatial position (Slatkin, 1985).

According to Solé-Cava (2001), populations of species threatened with extinction are frequently structured since, in general, environmental degradation leads to the formation of refuges (fragments) where small populations of these species persist but are unable to exchange genetic material with other plants located in unaltered areas. The *J. decurrens* populations examined here constituted refuges, each with a high level of structuring.

The results of this study may be useful in establishing conservation strategies of endemic species such as *J. decurrens* in the State of São Paulo. One possibility is to preserve the genetic variability found in the area of plant occurrence (*in situ* conservation) since local adaptations tend to favor the establishment of a species in its natural habitat. With the exception of the Mogi Guaçú population, which is located on the Mogi Guaçú Biological Reserve, all of the other populations are located on private lands that have experienced different degrees of human intervention. Since it is likely that in the near future these populations will cease to exist because of increased land use, we suggest that samples of the material analyzed here be preserved in a germplasm bank.

## Figures and Tables

**Table 1:** Identification, geographic location, number of individuals and genetic distances (Nei, 1978) for the *J. decurrens* populations studied in this work. The geographic distances (in km) and genetic distances (Nei, 1978) among the populations are shown above and below the diagonal, respectively.

Population	Longitude	Latitude	Altitude (m)	No. of samples	Distance between populations					
					MG	SC	PRAT	ITA	PP	ALT
Mogi-Guaçu (MG)	-47°10'	-22°15'	613.7	9	*****	66.01	174.0	190.0	175.0	137.0
São Carlos (SC)	-47°47'	-22°10'	780.0	8	0.0133	*****	122.0	152.0	172.0	126.0
Pratânea (PRAT)	-48°44'	-22°48'	740.0	17	0.0161	0.0139	*****	49.19	280.0	235.0
Itatinga (ITA)	-48°38'	-23°16'	607.4	16	0.0192	0.0217	0.0187	*****	320.0	273.0
Patrocínio Paulista (PP)	-47°17'	-20°40'	760.0	10	0.0220	0.0259	0.0230	0.0164	*****	46.66
Altinópolis (ALT)	-47°29	-21°03'	616.5	30	0.0457	0.0488	0.0419	0.0377	0.0267	*****
			Total	167						

**Table 2:** Primers used in this study and RAPD markers for 90 individuals of *J. decurrens.*

		Polymorphic bands		
Primers	Sequence (5'→ 3')	Total number of bands	Number of polymorphic bands	%
Operon A-01	CAG GCC CTT C	12	9	75
Bio-Synthesis Inc. 2G4-62	ACG AAC GCA CCA ATG AGC	9	8	88
Operon A-02	TGC CGA GCT G	10	8	80
Operon A-08	GTC ACG TAG C	11	6	54
Operon A-18	AGG TGA CCG T	8	2	25
Bio-Synthesis Inc. 3C11-3	TGC TTC GGG TAG CTC TTG C	5	5	100
Operon H-03	AGA CGT CCA C	7	3	42
Operon N-13	AGC GTC ACT C	6	6	100
Operon A-13	CAG CAC CCA C	7	4	57
Operon AV-15	GGC AGC AGG T	3	3	100
Total		78	54	-
Mean		7.8	5.4	69.2

**Table 3:** Primer combinations used and AFLP markers obtained for 90 individuals of *J. decurrens.*

	Polymorphic bands		
Primer combinations	Total number of bands	Number of polymorphic bands	%
E-ATC+M-ATC	29	13	44.8
E-ATT+M-ATG	30	12	40
E-ATG+M-AGT	73	38	52
E-AGT+M-ATT	73	32	43.8
Total	205	95	-
Mean	51.3	23.8	46.3

**Table 4:** Basic descriptive statistics for RAPD and AFLP analyses of populations of *J. decurrens* in the State of São Paulo. Hardy-Weimberg equilibrium was assumed. The mean sample size ( $\bar{n}$), number of observed alleles per locus (na) and percentage of polymorphic loci (P) are shown. The parameters were calculated with the POPGENE program (Yeh *et al.*, 1999).

		RAPD			AFLP	
Population	$\bar{n}$	na	P		na	P
Mogi Guaçú	9	1.1667	16.67		1.2683	26.83
São Carlos	8	1.1538	15.38		1.2293	22.93
Pratânea	17	1.1923	19.23		1.683	26.83
Itatinga	16	1.2308	23.08		1.2829	28.29
Patrocínio Paulista	10	1.1923	19.23		1.585	25.85
Altinópolis	30	1.6282	62.82		1.3317	33.17
Total	90	1.6923	69.23		1.4634	46.34

**Table 5:** AMOVA between and within populations of *J. decurrens* (n = 90 individuals/population) based on RAPD and AFLP results. DF, degrees of freedom, SQ, sum of squares, SQM, sum of mean squares, p, level of significance for the estimate of genetic variation based on 1000 permutations. PHI_st_ statistic, genetic variation estimated for sources of variation analogous to the Wright statistic F_ST_. The analyses were done using the programs AMOVA-PREP 1.01 (Miller, 1998) and WINAMOVA 1.04 (Excoffier, 1992).

Source of variation (RAPD)	DF	SQ	SQM	Variance components	% of total variation	p	PHI_st_ statistic
Between populations	5	162.29	32.45	1.96	30.31	< 0.001	0.303
Within populations	84	378.99	4.1	4.51	69.69	-	-
Total	89	541.28					

Source of variation (AFLP)	DF	SQ	SQM	Variance components	% of total variation	p	PHI_st_ statistic

Between populations	5	283.80	56.76	3.33	26.34	< 0.001	0.263
Within populations	84	782.49	9.315	9.315	73.66	-	-
Total	89	1	066.29				
